# The Complexity of Skeletal Transverse Dimension: From Diagnosis, Management, and Treatment Strategies to the Application of Collaborative Cross (CC) Mouse Model

**DOI:** 10.3390/jfmk9010051

**Published:** 2024-03-14

**Authors:** Nezar Watted, Iqbal M. Lone, Kareem Midlej, Osayd Zohud, Obaida Awadi, Samir Masarwa, Ali Watted, Eva Paddenberg, Sebastian Krohn, Christian Kirschneck, Peter Proff, Fuad A. Iraqi

**Affiliations:** 1 Center for Dentistry Research and Aesthetics, Jatt 45911, Israel; nezar.watted@gmx.net (N.W.); awadi.obaida@gmail.com (O.A.); sameer.massarwa@gmail.com (S.M.); 2 Department of Orthodontics, Faculty of Dentistry, Arab America University, Jenin 919000, Palestine; 3 Gathering for Prosperity Initiative, Jatt 45911, Israel; 4 Department of Clinical Microbiology and Immunology, Sackler Faculty of Medicine, Tel Aviv University, Tel Aviv 6997801, Israel; iqballone@mail.tau.ac.il (I.M.L.); kareemmidlej@mail.tau.ac.il (K.M.); osaydzohud@mail.tau.ac.il (O.Z.); 5 Department of Cranio-Maxillo-Facial Surgery, University of Hannover, 30625 Hanover, Germany; ali.watted@gmx.de; 6 Department of Orthodontics, University Hospital of Regensburg, University of Regensburg, 93053 Regensburg, Germany; eva.paddenberg@ukr.de (E.P.); sebastian.krohn@klinik.uni-regensburg.de (S.K.); peter.proff@klinik.uni-regensburg.de (P.P.); 7 Department of Orthodontics, University of Bonn, 53111 Bonn, Germany; christian.kirschneck@uni-bonn.de

**Keywords:** skeletal transverse dimension (STD), orthodontic therapy, occlusal relationships, centric relation (CR), central occlusion (CO), orthopedic maxillary expansion (OME), surgically assisted maxillary expansion (SARME), collaborative cross mouse model

## Abstract

This study investigates the significance of skeletal transverse dimension (STD) in orthodontic therapy and its impact on occlusal relationships. The primary goal is to enhance understanding and promote the integration of transverse skeletal diagnostics into routine orthodontic assessments. To achieve this aim, the study employs a comprehensive approach, utilizing model analysis, clinical assessments, radiographic measurements, and occlusograms. The initial step involves a meticulous assessment of deficiencies in the maxilla, mainly focusing on transverse dimension issues. Various successful diagnostic methods are employed to ascertain the type and presence of these deficiencies. Furthermore, the study compares surgically assisted maxillary expansion (SARME) and orthopedic maxillary expansion (OME) in addressing skeletal transverse issues. Stability assessments and efficacy analyses are conducted to provide valuable insights into the superiority of SARME over OME. The findings reveal that proper evaluation of STD is crucial in orthodontic diagnosis, as overlooking transverse dimension issues can lead to complications such as increased masticatory muscle activity, occlusal interferences, and an elevated risk of gingival recession. Surgically assisted maxillary expansion emerges as a more stable solution than orthopedic methods. In conclusion, incorporating skeletal transverse diagnostics into routine orthodontic assessments is imperative for achieving optimal occlusal relationships and minimizing negative consequences on dentition, periodontium, and joints. The study emphasizes the significance of accurate three-dimensional assessments and recommends the consideration of SARME over OME for addressing skeletal transverse deficiencies. Finally, the Collaborative Cross (CC) mouse model is also a novel mouse model for studying complex traits. Exploring the Collaborative Cross mouse model opens avenues for future research, promising further insights into transverse skeletal issues in orthodontics.

## 1. Introduction

Static and functional occlusal relationships are the well-defined objectives of orthodontic therapy. To accomplish Andrews’ six keys to proper dental occlusion [1], the jaws need to be positioned in centric relation (CR)/central occlusion (CO) and appropriately proportioned in three planes of space. Orthodontists have well-established techniques when analyzing the skeletal association between the maxilla and mandible in both sagittal and vertical planes [2,3,4,5,6]. Although multiple analyses exist for the transverse dimension, their inclusion in conventional orthodontic diagnosis is not widely acknowledged [3,6,7].

When the jaws are not in perfect alignment, the dentition will try to make up for it in the sagittal dimension, which can lead to anterior teeth that are too proclined or retroclined. Tooth structures may either emerge into a crossbite or reposition themselves to prevent one when the jaws do not relate appropriately in the transverse dimension, typically caused by a maxilla width defect [7,8].

Transverse tooth compensations have been visually represented in the prosthodontic literature utilizing a crosshatch arch that passes through the buccal and palatal cusps of maxillary molars. We refer to this as the Wilson curve. The palatal cusps are situated beneath the buccal cusps, resulting in the maxillary molars exhibiting an exaggerated inclination to offset the limited width of the maxilla, resulting in a significantly exaggerated Wilson curve. Numerous papers discussing how CR/CO inconsistencies impact occlusion primarily address its impact on sagittal and vertical dimension diagnosis. The “plunging” palatal cusps depicted are thought to be the main points of contact that cause vertical condylar distraction at closure from CR, according to the literature. The maximal intercuspal position can be achieved by the patient’s pivot away from untimely contacts in the end molars when in a seated condylar position. Visual indicators of this vertical aspect of condylar distraction include the Panadent Condylar Position Indicator (CPI) and the SAM Mandibular Position Indicator (MPI) [9,10,11,12].

McNamara and McClatchey [13] state that “the orientation of the maxillary posterior teeth’s lingual cusps frequently lies below the occlusal plane”. Constriction of the maxilla followed by dentoalveolar adjustments, leading to a slight flaring of the maxillary posterior teeth, are frequently the cause of this prevalent appearance in patients with malocclusion. According to Bin Dakhil and Bin Salamah [14], an inflated Wilson curve along with a transverse maxilla deficit may lead to vertical displacement of the condyles in cases involving CR/CO inconsistencies. The authors have concluded that the vertical condylar shift from CR to maximal occlusion centers around the enlarged Wilson curve and the plunging palatal cusps when there is no posterior crossbite. Moreover, extrapolating this statement implies that a significant portion of the CR/CO gap can be reduced when the transverse skeletal dimension is standardized, the Wilson curve becomes leveled, and the arches are harmonized.

### 1.1. Transverse Deficiency and Working/Nonworking Interferences

An inflated Wilson curve has long been seen as a prosthetic maxim, increasing the possibility of both working and non-working side interferences. Research has indicated a connection between higher masticatory muscle activity and posterior occlusal contacts or interferences [15,16]. In previous experiments where these interferences are eliminated, the closure musculature’s activity is diminished [16,17]. Furthermore, a research endeavor that artificially introduced deflective interferences noted a heightened muscle engagement [18]. The findings indicate that it is wise to flatten the Wilson curve and normalize the transverse jaw relationship to rule out the possibility of excursive posterior interferences or contacts.

### 1.2. Transverse Deficiency and the Periodontium

While discussing the impact of concealed skeletal transverse issues through dental expansion, it is crucial to highlight the associated risk of gingival recession, as demonstrated by McNamara [19] in orthodontic patients with small maxillas. Notably, the transverse treatment envelope presents constraints, primarily allowing for the enlargement of dentures rather than a comprehensive alteration of the skeletal structure [20]. This limited movement becomes a critical factor, particularly in the thin layer of cortical bone in the alveolus.

## 2. Materials and Methods

All human samples presented in this study were assessed and treated according to current guidelines and following the Ethics Committee of the University of Regensburg ethics and regulations, the committee reviewed and approved this research project and study design with approval number 19-1596-101 (dated 13 November 2019). All patients were assessed and treated at the Orthodontic Research Center based on Jatt, Israel.

### 2.1. Diagnosis

**Figure 1:**
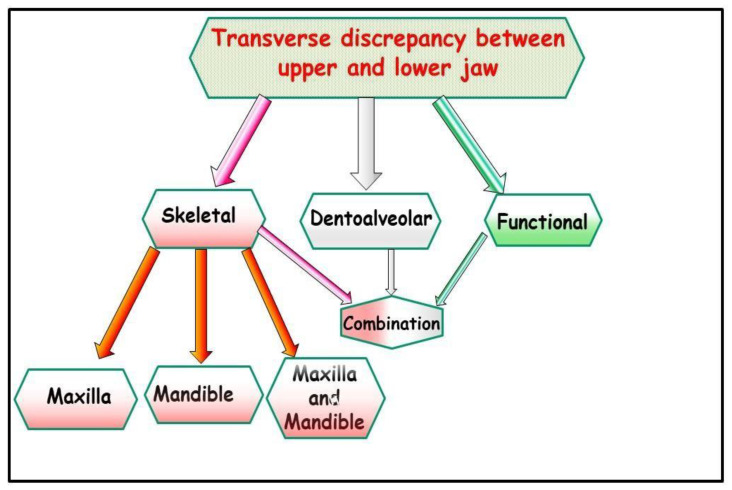
Schematic representation of the various transverse discrepancies between the upper and lower jaw. There can be several causes for these transverse inconsistencies.

There are several causes for the transverse discrepancy between the upper jaw and lower jaw, indicating a narrow upper jaw, bilateral crossbite, unilateral crossbite with or without facial asymmetry (Figure 1).

**Figure 2:**
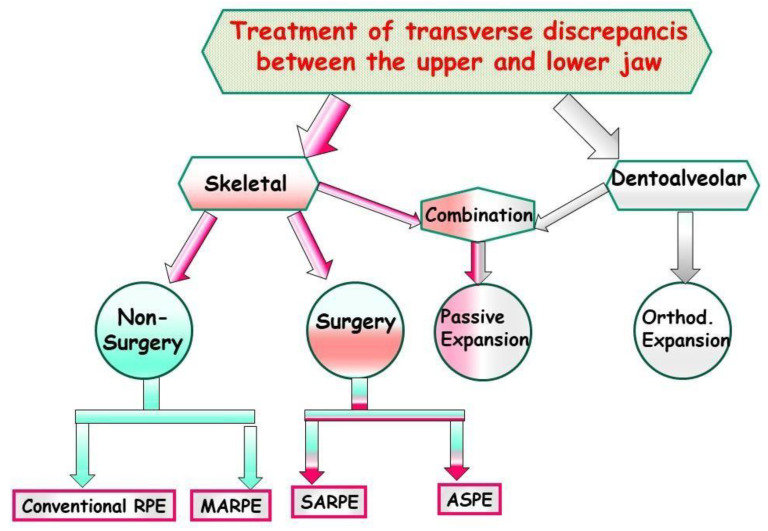
Schematic representation of different treatment methods for transverse discrepancies between the upper and lower jaw.

Depending on age, growth stage, goal, and amount of expansion, there are different methods available for expanding the narrow maxilla (Figure 2).

### 2.2. Narrow Upper and Lower Jaw

**Figure 3:**
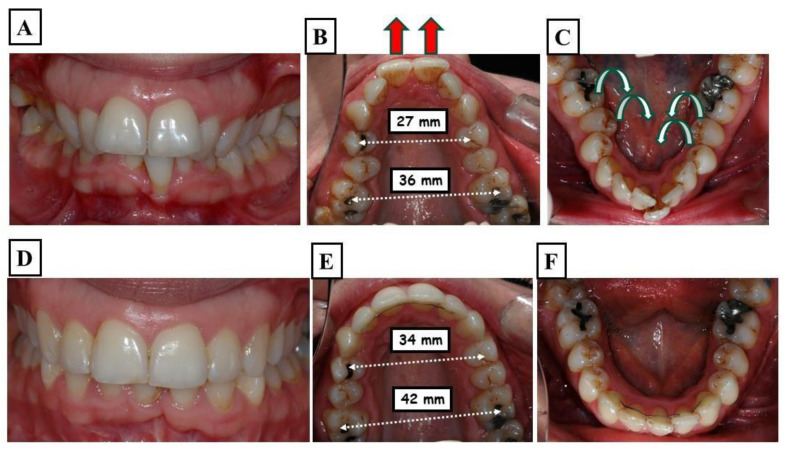
A demonstration of a patient with a very narrow upper and lower jaw. The figure shows the situation before the start of treatment—proclination of the upper front teeth with an increased overjet (**A**), the upper jaw width in the molar region is 36 mm, and the molars are tilted lingually, the proclination of the upper incisors (re d arrows) caused an additional enlargement of the overjet (**B**), and the lower jaw has adapted to the shape of the upper jaw, showing severe crowding and lingual tilting of the molars (**C**). (**D**–**F**) show the situation at the end of treatment, including, stable functional occlusion, well-formed and levelled upper and lower jaws, respectively. Harmonious dental arches in all three dimensions.

For the mandibular posterior teeth, this compensation usually entails lingual tilting, which results in an overly negatively inclined mandible. Furthermore, the maxillary posterior teeth are tipped facially. Afterward, it is said that these teeth have an overly optimistic slope (Figure 3A–F).

### 2.3. Dental Expansion

**Figure 4:**
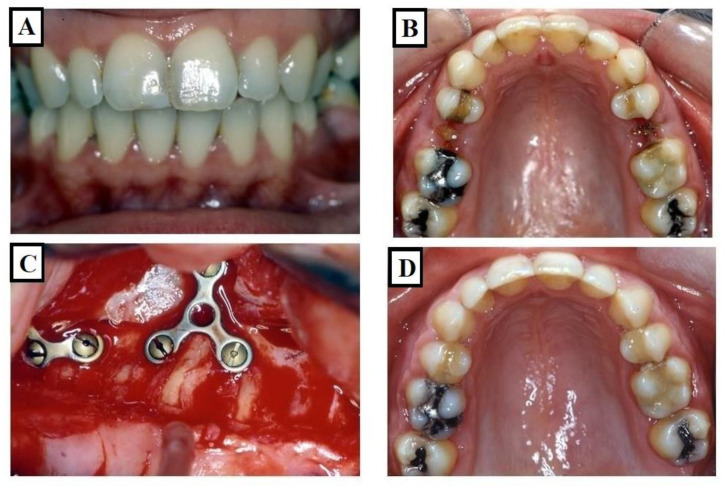
Images of a patient who underwent dental expansion of the upper jaw and an upper jaw osteotomy. (**A**,**B**) show the case before the treatment, while (**C**,**D**) show during the operation on the upper jaw; fissures in the bone were observed. This is associated with dental and alveolar expansion.

Research by Harrell [21] Davies [22] and Saravanan [23] has underscored that individuals at risk of periodontal disease exhibit a better long-term prognosis when working and nonworking interferences are eliminated. In the context of transverse deficiencies, the reduction in buccal alveolar bone associated with skeletal expansion may lead to weakened gingival tissues and an increased risk of gingival recession (Figure 4A–D). Thus, normalizing the transverse jaw connection not only eliminates an exaggerated Wilson curve and nonworking interferences but may also be advantageous for adult individuals susceptible to periodontal concerns, potentially preventing such risks in younger patients. By elucidating the relationship between transverse deficiencies, dental expansion, and the subsequent risk of gingival recession, this study emphasizes the importance of considering periodontal implications in orthodontic treatment planning.

### 2.4. Transverse Deficiency and the Airway

**Figure 5:**
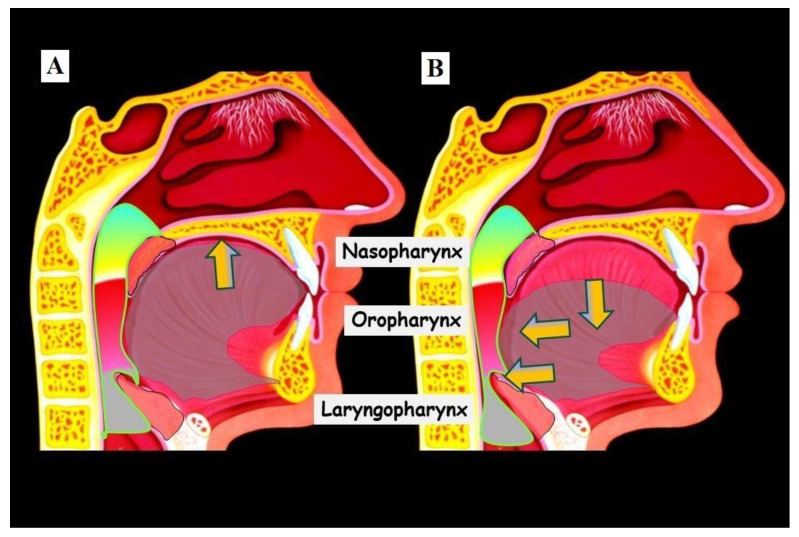
Illustration of tongue position and airways. (**A**) shows the physiological tongue position in a normally wide upper jaw on the palate (The yellow arrow indicates the direction of position on the palate). In this tongue position, the airways are most open, and usually, there are no breathing disturbances through the nose. (**B**) shows the case of a narrow upper jaw; the tongue rests on the floor of the mouth (Yellow arrow directed caudally), pushing the tongue posteriorly (Yellow arrows directed posteriorly). This constriction particularly affects the airways in the area of the laryngopharynx, leading to impaired nasal breathing.

**Figure 6:**
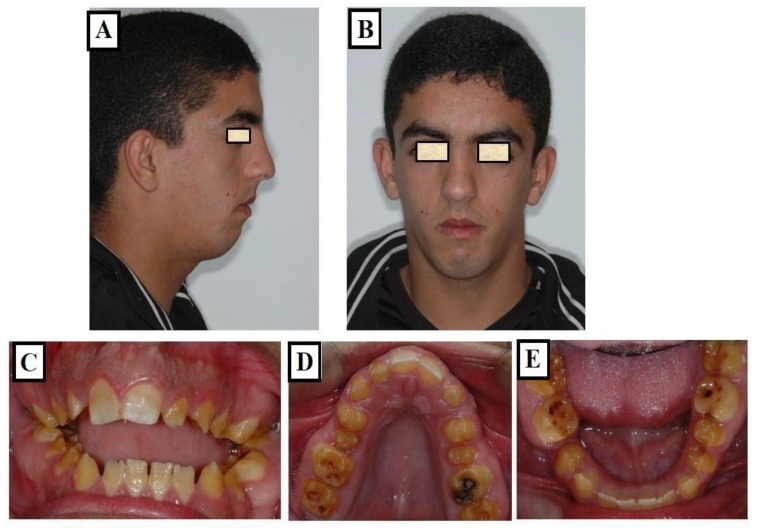
Images of a patient with Long Face Syndrome. The following figures show the characterizations including a significant retrusion of the lower jaw (**A**,**B**), skeletal and dentoalveolar open bite (**C**), narrow upper jaw, deep palate (**D**), and broad lower jaw (**E**).

The narrow maxilla and constricted nasopharyngeal airway are further suggested to be related by Ricketts’ description of “adenoid facies” [24]. Children that have nasal passage impairments, according to Ricketts, mostly breathe through their mouths. The pressure exerted by the circumoral musculature functions without resistance because the glossal structure, which is located at the base of the mouth to enhance air circulation, lacks the capacity to sustain the developing palate. As teeth erupt, the palate narrows, and an accentuated Wilson curve appears. In addition to developing a retruded, high-angle mandibular shape, the patient may also have an increased risk of sleep apnea due to the low position of the tongue in the mouth [25] (Figure 5A,B and Figure 6A–E).

### 2.5. Methods of Transverse Diagnosis

The temporomandibular joints, muscles, periodontal tissue, and respiratory passage might all suffer in a susceptible patient with a transverse insufficiency brought on by a narrow maxilla. As orthodontists, our objective needs to be creating skeletal connections and an optimal dental closure as close to ideal as feasible. This will reduce the likelihood that any occlusion-related inconsistencies would exacerbate the negative consequences on the dentition, periodontium, or joints. An accurate skeletal and dental diagnostic in all three spatial dimensions is required to accomplish this. This portion describes three approaches to transverse dimension diagnosis: dental casts, cone-beam computed tomography (CBCT), and classical cephalometry. Our goal in providing an explanation of all three approaches is to enable readers to use a transverse skeletal diagnostic in their practice regardless of the technological level at their disposal. We do not recommend any one of these approaches over the others. Normalizing the transverse dimension requires consideration of ideal treatment goals, regardless of the approach.

Study models should be used to assess the arch’s form and shape. This will make it possible to take precise measurements that assess the maxilla’s transverse deficit. Indexes for such lateral measurements have been proposed by several authors [26]. These indexes aid in the diagnosis of STD, but because they are population-specific, they cannot be fully relied upon [27]. A study cast must be used to further assess the transverse tooth inclination variations and arch symmetry. When there is no lateral displacement or chin asymmetry, bilateral cross bite occlusion might be observed. If this is discovered, a transverse disparity of this kind can be classified thanks to the subject’s examination models and comprehension of the sagittal connection. To ascertain if the transverse insufficiency is absolute or relative, examination of the patient study castings is once more necessary. The transverse irregularity is considered relative when the posterior teeth would correctly align and occlude (assuming correct teeth arrangement) with the canines are positioned in Class I occlusion in this scenario, if the posterior teeth do not exhibit the proper transverse cusp fossa relationships in centric relation [28,29]. For example, casts (arches) that articulate into a Class I canine connection eliminate a posterior crossbite that occurs in certain Class III malocclusions. Once more, this transverse disparity is categorized as relative. Still, a crossbite is considered an absolute transverse disparity if, following the articulation of a Class I canine connection in the castings, it persists [28]. Should an absolute transverse discrepancy exist, study casts are utilized to ascertain the discrepancy’s extent and origin (skeletal or dental). Initial investigation should focus on posterior dental compensations within the cast. The permanent first molars will exhibit changes in their transverse axial inclination, which are typically caused by an excessive amount of torque applied to either the mandibular or maxillary buccal crowns in relation to the frontal plane [28].

It is sufficient to make a rough estimate or use the American Board of Orthodontics (ABO) measurement tool. An ABO gauge is used to define a transverse occlusal plane between the first molars on the left and right. Should the molar under examination exhibit a lateral axial tilt perpendicular to the plane under examination, the gauge ought to make contact with the buccal and lingual cusps. The buccal or lingual cusps’ displacement from the transverse occlusal plane will be evident if they deviate from this perpendicular inclination. With the ABO gauge, 1 mm increments of guess are possible. The gap between the lingual and buccal cusps of molars with average width is 5–6 mm. Therefore, 10° of buccolingual inclination corresponds to a 1 mm displacement from the transverse occlusal plane. This technique can also be used to assess the mandibular molars’ buccolingual tilt [28]. The molar inclination of people who have regular sagittal and lateral occlusion was examined by the contributors of the Iowa Facial Growth Study [30]. The molar inclination of participants, who ranged in age from 7 to 26, was found. Subjects’ maxillary molars, who were seven years old, had an average buccal crown inclination of 10° ± 4° [30]. A lingual crown inclination of 10° ± 5° was seen in the mandibular molars of the same patients [30]. A greater perpendicular inclination of the mandibular and maxillary molar teeth to the transverse occlusal plane was seen as a consequence of later growth [28]. An approach involving tooth counting has been proposed to distinguish between dental and skeletal disparities [31]. A disparity in the crossbite is considered skeletal if it involves two or more posterior teeth [32]. The rule is helpful because of its simplicity, yet it is also deceptive. Severe skeletal transverse disparities may exist even in the absence of posterior teeth in a crossbite, concealed by posterior dental compensations. A dental etiology for the transverse discrepancy is plausible if up righting the molar in the cast improves the posterior transverse interarch relationship (i.e., eliminates the transverse compensations). Then, dental movement alone could be used as a form of therapy. A skeletal basis for the disparity is far more plausible if the posterior transverse interarch relationship worsens in the same instance [28].

As the most reliable and most accessible method, Betts et al. [33] recommend that posteroanterior (PA) cephalograms are used for the identification and assessment of transverse anomalies of structural etiology within the lower jaw and upper jaw. In order to determine two measurements of transverse insufficiency in the upper jaw, Betts et al. [34] use a collection of cephalometric markers that Ricketts [35] gives, the maximum longitudinal difference index and the maximum longitudinal width difference. However, there are criticisms of these approaches. The apical and dental bases, for example, are distantly located from the bone landmarks used to quantify the transverse discrepancy between the maxillary and mandibular [36]. With additional measurements needed for the examination of maxillary/mandibular transverse discrepancy, Ricketts’ Rocky Mountain methodology [35] was designed to determine comparative standards among particular radiographic points. With the use of such landmarks, the frontal ateralfacial lines as well as the measurable widths of the mandible and maxilla can be measured. The following definition applies to these widths: the right antegonial tubercle (AG) and the left antegonial tubercle (GA) align with jugale left (JL) and jugale right (JR), while the width of the mandible is the measurement between these two points. Assembled from the orbitales right (OR) and left (OL) to the points AG and GA, accordingly, the frontalateral facial lines are the lateral lines. By first calculating the transverse difference index between the maxilla and mandible, and the differential width between the maxilla and mandible, these cephalometric points can serve as references to assess the transverse maxillary discrepancy. The latter of them is only the displacement that is measured, in millimeters, between JL and JR and the frontolateral facial line, correspondingly, following a trajectory from the frontolateral facial lines through JR and JL. This is a side-by-side independent measurement that is contrasted with the standard value of 10 ± 1.5 mm. A transverse mismatch between the lower jaw and upper jaw is indicated by a value larger than 10 mm. By adding up the recorded quantities on both sides that are more than 10 mm, the overall transverse deficiency is calculated. This is an effective way to show the overall difference and show which side is more deficient. However, this method can be misconstrued in the presence of asymmetry in the mandible since it is unable to accurately identify which jaw is affected [34].

In contrast, the transverse difference index between the maxilla and mandible subtracts the real distance between the maxilla and mandible from the projected difference based on the individual’s age. The real maxillomandibular discrepancy is determined by subtracting the measured AG-GA from the measured JR-JL, whereas the expected difference is the age-specific expected AG-GA distance minus the age-appropriate expected JR-JL distance. In an adult patient, a maxillomandibular transverse differential index of more than 5 mm suggests the need for surgical expansion. This method allows one to determine whether the jaw is excessive or deficient in addition to quantifying the overall disparity [34].

In order to accurately study the craniofacial region, more current methods have been developed, such as three-dimensional (3D) imaging, which has made it possible to visually assess the spatial relationships between the components of the jaws [37]. Cone-beam computed tomography (CBCT) imaging permits the cross-sectional analysis of three-dimensional depictions of the apical structures. The nature and position of inconsistencies as well as asymmetries can be assessed more precisely and thoroughly by a doctor using these images [36]. In addition to having significant diagnostic value for evaluating areas of concern, targeted, localized transversal radiography cuts of CBCT scans also have significant diagnostic potential for the transverse dimension of the craniofacial. With more CBCT imaging becoming available, it is pertinent and advantageous to ascertain whether this technology can enhance STD diagnosis or if its utility is limited to pinpoint accuracy [38].

## 3. Results

**Figure 7:**
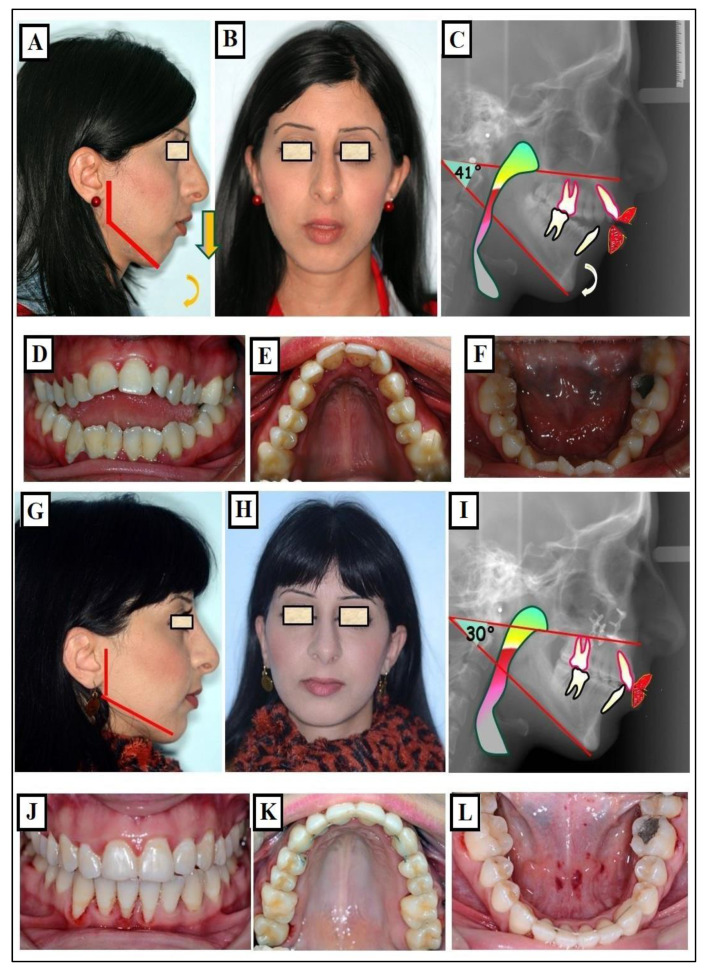
Images of a patient with Long Face Syndrome. (**A**,**B**) show the retrognathic lower jaw. The interbasal angle between the maxilla and mandible is 41° increased by 18° (the normal value is 23°), A significantly enlarged jaw angle or gonion angle (red lines 7a) resulting in a skeletal open bite and dentoalveolar open bite. The narrowed airways (The colored space) cause breathing disturbances (**C**,**D**), narrow upper jaw, deep palate (**E**), and broad lower jaw (**F**). The status of the same patient after a combined orthodontic and surgical treatment is presented. Harmonization of the face in the vertical dimension with maxilla impaction to correct the skeletal open bite as a result, there was a reduction in the jaw angle (red lines) (**G**,**H**). The expansion of the upper jaw and surgical impaction led to autorotation of the mandible and closure of the skeletal open bite, the intermaxillary angle decreased from 41° to 30° due to the autorotation of the mandible (red lines) (**I**). As a result, there is an improvement in tongue position and airway expansion (colored area) (**I**). A well-expanded and formed upper and lower jaw is achieved (**J**–**L**).

Due to the narrow palate and incorrect tongue position, nasal breathing disturbances occurred, negatively impacting further growth. A recent study found that individuals treated with fast palatal extension who had transverse limitations resulting from a narrow maxilla had an 8–10% increase in upper airway volume [39]. An increase in the upper airway’s volume was also noticed in individuals exhibiting dental posterior crossbites who underwent treatment involving the widening of the palate [40]. Palatal expansion reduced nasal resistance and enhanced nasal breathing, according to Oliveria de Felippe et al. [41]. Any increase in the airway’s volume as a result of widening of the palate to maximize the jaws’ width could be extremely beneficial for an individual’s general growth and development, even if further research in this field is undoubtedly required (Figure 7A–L).

### 3.1. The Causes of the Discrepancy in Jaw Widths Can Be

#### 3.1.1. Functional

**Figure 8:**
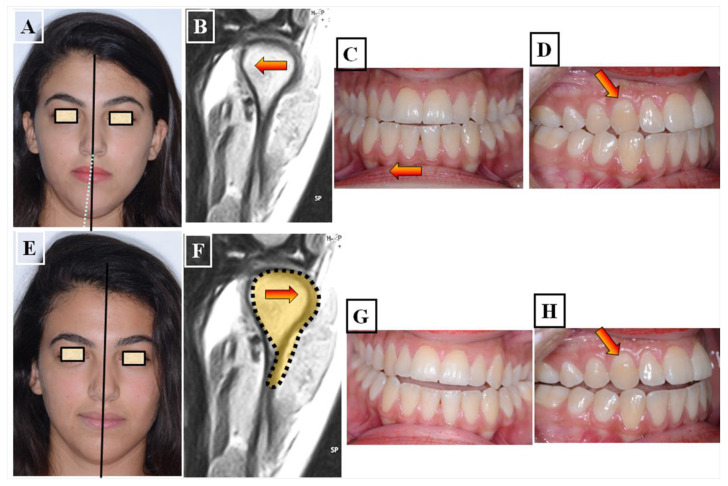
Determination of the centric condyle position and thus centric occlusion after using a flat occlusal splint for decoupling the occlusion for 2–3 weeks. Situation before treatment and before using the occlusal splint, (**A**–**D**). Forced bite with crossbite to the right in the area of the canines 13 and 43 (arrow) with facial asymmetry to the right (white dashed line), habitual condyle position with habitual occlusion (The forced bite direction resulted in a change in position of the condyles (red arrow). The status of the patient after 3 weeks of using the occlusal splint. The forced bite has been eliminated, Instead of a crossbite on the canines, there is an edge-to-edge bite (red arrow). There is no facial asymmetry in the centric condyle position, the centralization of the condyles resulted in a new position of the condyles (red arrow) (**E**–**H**).

In this situation, two different occlusions with two different condyle positions can be observed—a habitual occlusion with a habitual condyle position and a centric occlusion with a centric condyle position. Typically, facial asymmetry is observed in habitual occlusion or the habitual condyle position. Manual functional analysis can achieve the centric condyle position with the corresponding change in occlusion. This helps reduce or completely eliminate facial asymmetry. A dentoalveolar malalignment is often involved in most cases (Figure 8A–H).

#### 3.1.2. Dentoalveolar

**Figure 9:**
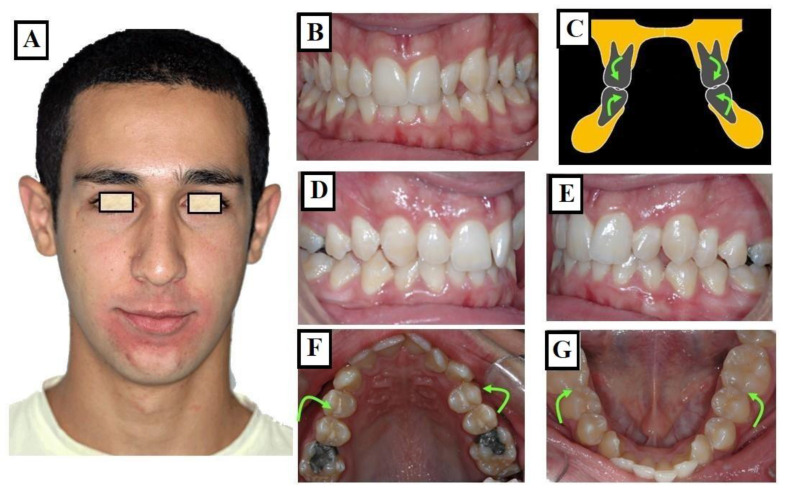
Narrow upper jaw due to dentoalveolar tilting towards the palatal side. The lower jaw dental arch has adapted to the upper jaw through lingual tilting, and no asymmetry is present. Narrow long face without asymmetry (**A**) with a narrow upper jaw due to dental tilting towards the palate (green arrows) as shown in (**C**,**F**). The lower dental arch is narrow and has adapted to the upper jaw shape due to dental tilting towards the lingual side (**C**,**G**). Due to the clinical adjustment of both dental arches to each other, no clinical crossbite is visible (**B**,**D**,**E**).

In this situation, it involves a dental misalignment in the transverse dimension without affecting the centric condyle position. There is a discrepancy in the transverse dimension, and the cause lies in the upper jaw. In these cases, no facial asymmetry is observed (Figure 9A–G).

#### 3.1.3. Skeletal

**Figure 10:**
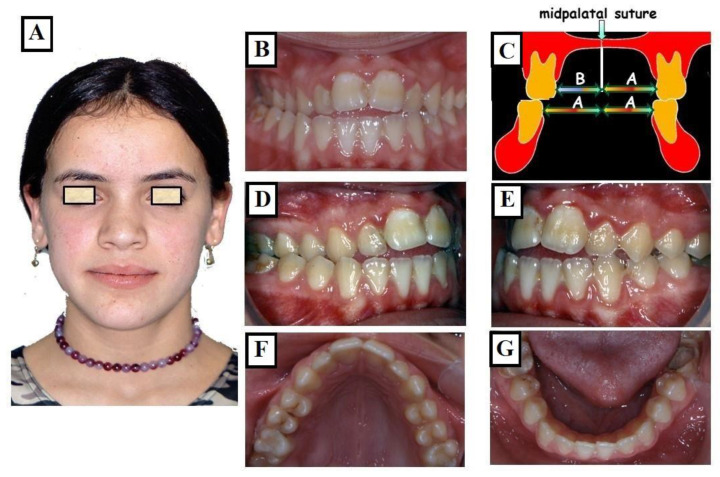
Transverse discrepancy between the upper jaw and lower jaw with a right-sided crossbite without facial asymmetry (**A**), unilateral skeletal crossbite on the right side due to a narrow upper jaw (**B**–**E**). The right half of the upper jaw—arrow B in (**C**,**F**)—is narrower than the left half of the upper jaw—arrow A in (**C**,**F**). The lower jaw is well-shaped (**G**).

**Figure 11:**
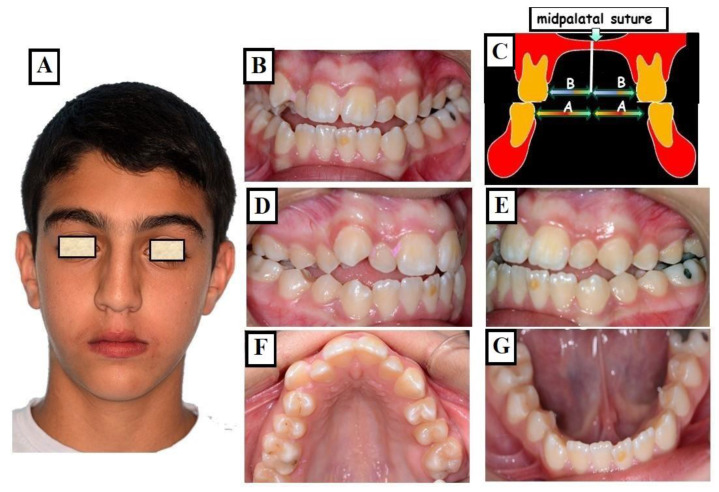
Transverse discrepancy between the upper jaw and lower jaw without facial asymmetry (**A**), crossbite on both right and left sides (**B**,**D**,**E**). The overall upper jaw is narrow (**C**,**F**), and the lower jaw is well-formed (**G**), the width of the maxilla, which consists of two halves that are equally wide (C arrows 2 × B), is narrower than the width of the mandible (C arrows 2 × A) partially adapting to the width of the upper jaw.

This skeletal dysplasia can occur in the upper jaw (narrow upper jaw base). In this situation, the upper jaw can be narrow on one side of the jaw, while the other half is normal (unilateral crossbite) (Figure 10A–G), or the entire upper jaw base is narrow. In these situations, no facial asymmetries are observed (Figure 11A–G).

**Figure 12:**
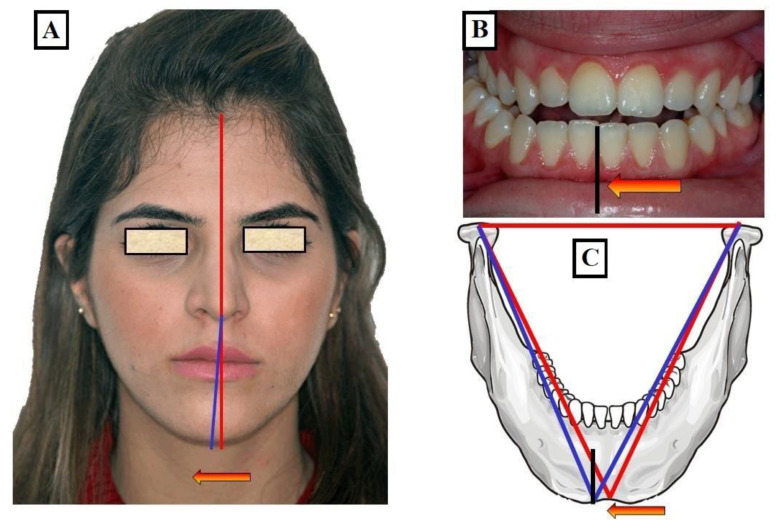
Transverse discrepancy between the upper jaw and lower jaw with facial asymmetry to the right deviation of the mandible to the right (red arrow, blue line) from the midline of the face (red line) (**A**) with a crossbite to the right (Black line and red arrow) (**B**), a minor skeletal or growth-related deviation of the lower jaw (red arrow, blue line) (**C**).

**Figure 13:**
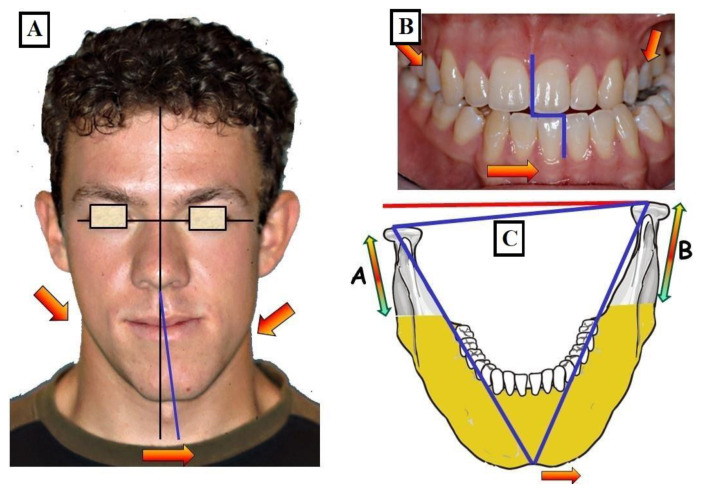
A significant transverse discrepancy between the upper jaw and lower jaw with facial asymmetry to the left deviation of the mandible to the left (red arrow, blue line) from the midline of the face (black line) (**A**) and a crossbite to the left (red arrow) with a mandibular midline shift to the left (blue line) (**B**), a pronounced skeletal or growth-related deviation of the lower jaw. This phenomenon is referred to as condylar hyperplasia. This deviation (blue triangle in relation to the red line, red arrow) is due to differences in the temporomandibular joint condyles (arrows A and B) (**C**).

When the transverse discrepancy is skeletal and originates from the lower jaw, facial asymmetry due to the lower jaw deviation towards the crossbite side can be observed. This asymmetry is typically a growth disorder in the condylar region of the lower jaw (Figure 12A–C). These growth disorders can be referred to as condylar hyperplasia when they reach a certain degree, resulting in pronounced facial asymmetry due to the lower jaw deviation to the opposite side (Figure 13A–C). In not uncommon cases, both the mandible and the maxilla are involved in this transverse discrepancy.

#### 3.1.4. Combination

**Figure 14:**
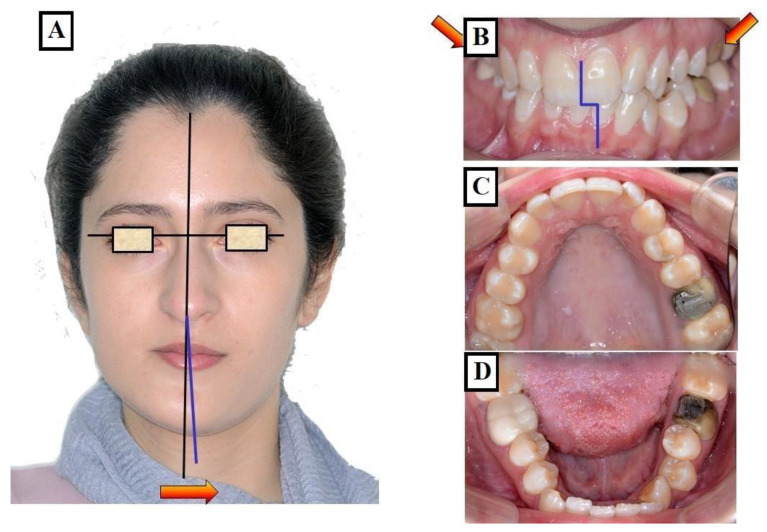
Transverse discrepancy between the upper jaw and lower jaw with facial asymmetry to the left deviation of the mandible to the left (red arrow, blue line) from the midline of the face (black line) (**A**), and a crossbite to the left (red arrow) with mandibular midline shift to the left (blue line) (**B**). The causes of this jaw discrepancy are functional, dentoalveolar, and skeletal (**C**,**D**), with a pronounced skeletal or growth-related deviation of the lower jaw due to condylar hyperplasia.

In this malformation, all the above-mentioned reasons for this transverse discrepancy can be responsible (Figure 14A–D).

Stability depends on early and precise identification and treatment for any dentofacial deformities including transverse deficit [34]. The initial step is to ascertain the kind and presence of deficiencies in the maxilla. Since there are fewer soft tissue alterations resulting from maxilla hypoplasia in the transverse direction, skeletal transverse dimension (STD) assessment poses a greater challenge than vertical or sagittal discrepancy assessment [34,42]. When distortions are limited to the anterior–posterior or vertical, these soft tissue alterations are far more common [34]. There is a large body of literature outlining the methods and standards used to diagnose maxillary insufficiency. Assessment of models, observations in clinical settings, radiographic assessments, and occlusal diagrams have all proven successful in producing accurate assessments [42].

**Figure 15:**
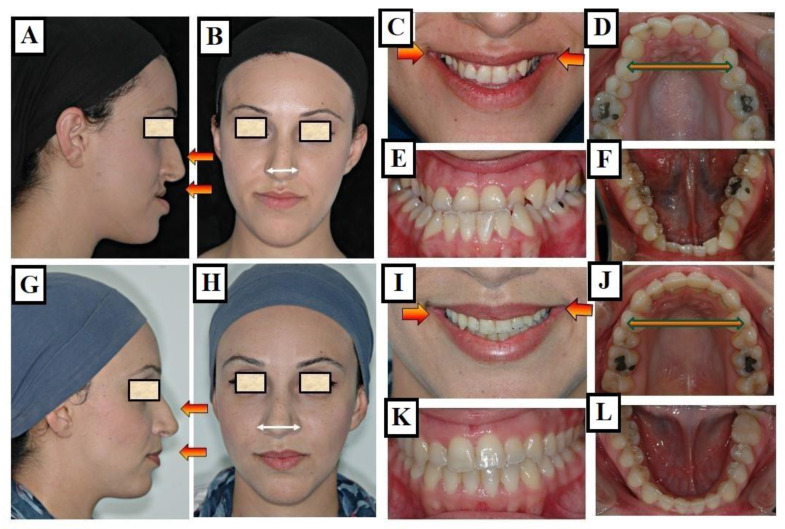
Patient with class III dysgnathia and midface hypoplasia. Patient’s condition before treatment leading to flattening of the paranasal region (red arrows), the width of the nose is narrow compared to the distance between the inner corners of the eyes (white arrow) (**A**,**B**) and darkening of the corners of the mouth (red arrows) (**C**), with a narrow maxilla between the first premolars (yellow arrow) and crossbite (**D**–**F**). Patient’s condition after treatment, harmonization of the face and paranasal structures (red arrows, white arrow), and improvement of the smile at the corners of the mouth (**G**–**I**). Formation of dental arches and establishment of a physiological occlusion (**J**–**L**).

The configuration and balance of the maxillary arch, the contour of the palatal vault, occlusion, the primary respiration pattern (i.e., nasal or oral), and the width of the oral passage during grinning were all assessed for clinical purposes. A transverse insufficiency in the maxilla can be detected by hollowing out the paranasal area, widening the buccal corridors excessively, deepening the nasolabial region, or reducing the width of the alar bases (Figure 15A–L). The modest soft tissue alterations linked to sexually transmitted diseases could make diagnosis more challenging. Therefore, the primary symptoms are pronounced crowding, rotation, or buccal/palatal displacement of the teeth, lateral/medial tooth misalignment, crossbite on one or both sides, an elevated palatal vault, and occlusions exhibiting an hourglass or V-shaped pattern. One of the causes of sexually transmitted diseases is mouth breathing. As a result, in otolaryngology practice, the aforementioned findings are sometimes noticed. Patients with STDs need to be assessed for mouth breathing and referred as needed to the relevant specialty. Excessive vertical dimension of the maxilla, coupled with a mandibular prognathic relationship, Apertognathic malocclusion (skeletal and/or dentoalveolar open bite), and corrected cleft palate are examples of dentofacial malformations linked to STDs. A physician can initially diagnose transverse insufficiency using these visual cues [34,43,44]. After closure, the mandibular shift needs to be evaluated. A frontal face examination may reveal lateral chin deviation; if this is the case, the underlying cause must be found. This can be the result of real bone asymmetries or a functional movement away from centric relation. If there is any doubt about the lateral displacement, it is best to temporarily disarticulate the occlusion for a period of one to two weeks, then have another examination done. Bite plates can be used for this. Here, the patient’s compliance could be in question, especially in the case of younger children. In this situation, the occlusion can be disarticulated, and a lateral displacement can be examined by inserting and gently activating a fixed (Hyrax) expander. It is imperative to notify all relevant stakeholders, including parents and patients, that therapy perhaps not definitively initiated unless the presence or absence of a lateral shift has been confirmed. Unilateral chin and crossbite asymmetry is used to suggest real unilateral skeletal asymmetry if a lateral shift is not observed [28].

Rapid Maxillary Expansion (RME)—or Rapid Palatal Expansion (RPE)—was introduced in the 1860s by E. Angle for the treatment of maxillary constriction and consisted of a shaft with tubular nuts that was rotated using a wrench made from a dime. To date, new technologically advanced tools have been introduced, but the final aim remains the same.

### 3.2. Expanding the Narrow Maxilla

#### 3.2.1. Passive Expansion

**Figure 16:**
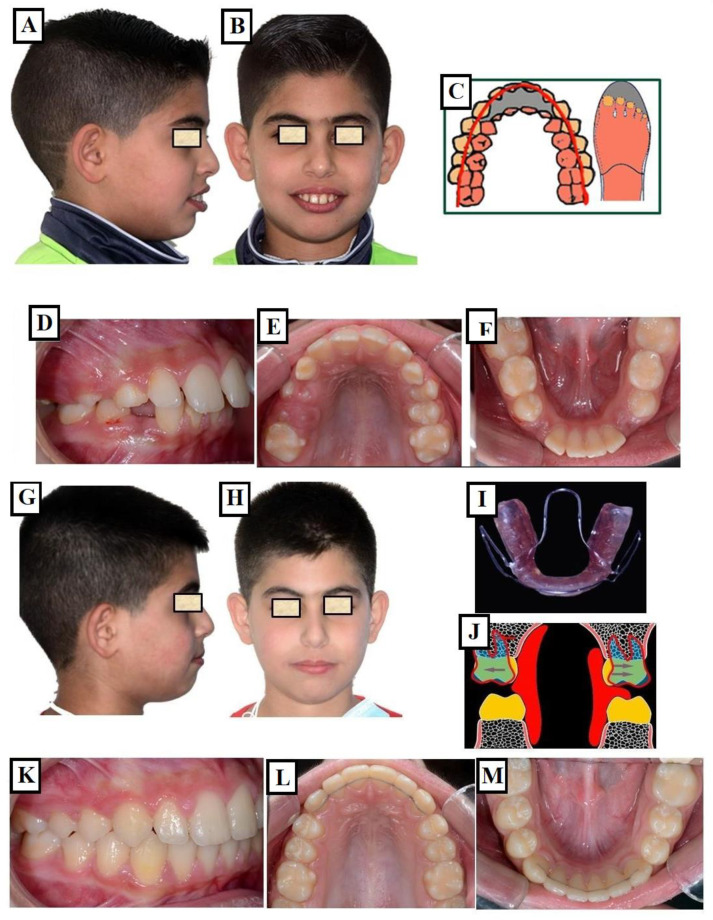
Treatment of class II with functional orthopedic appliance through passive expansion of the maxilla. Patient with class II dysgnathia (**A**,**B**). Typically, there is a transverse discrepancy between the upper and lower jaw before treatment following the principle of the pantoffel effect (**C**). The upper jaw is relatively narrow for the lower jaw in the therapeutic goal occlusion (**D**–**F**). The status of the patient after treatment (**G**,**H**,**K**–**M**) with a Bionator—a functional orthopedic appliance (**I**). There is passive expansion of the jaw due to functional forces and control of tooth eruption buccally (transparent blue teeth with arrows pointing buccally) (**J**).

This is a slow skeletal and dentoalveolar expansion. This method is used for growing patients and during the period of tooth eruption and without the use of force. The skeletal dysgnathy (e.g., in the treatment of class II dysgnathy, in which the maxilla is usually narrower than the mandible) is treated by using orthodontic appliances. By controlling the tooth eruption in a buccal direction with the appliance, the upper jaw is expanded (Figure 16A–M).

#### 3.2.2. Orthodontic Expansion

**Figure 17:**
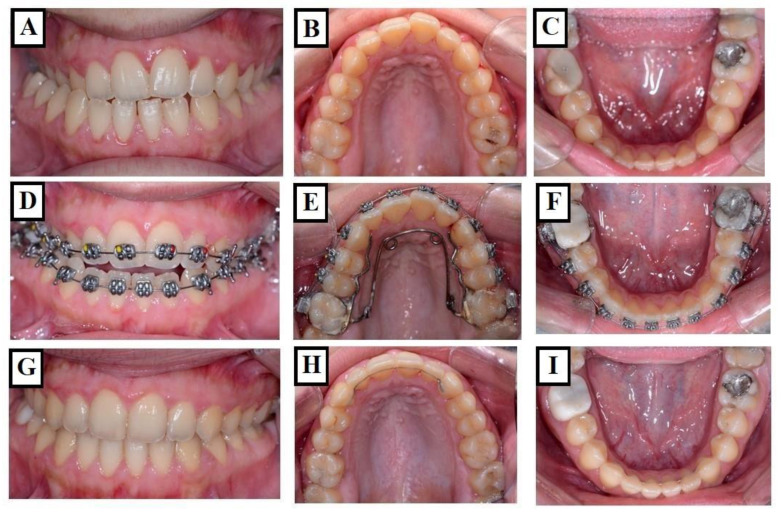
Orthodontic expansion using a fixed appliance—Quadhelix. (**A**–**C**): Before treatment, the upper jaw is narrow with a right-sided crossbite and well-formed mandible. A Quadhelix appliance was used for the expansion of the upper jaw (**E**) and during treatment (**D**–**F**). Situation after treatment (**G**–**I**).

This is a dentoalveolar expansion of the maxillary dental arch. This dentoalveolar movement is limited due to the anatomical conditions (bone and gingival thickness). By using continuous or discontinuous forces with the different active devices (fixed or removable appliances), the posterior teeth are moved buccally (Figure 17A–I).

#### 3.2.3. Orthopedic Expansion

This expansion is a skeletal expansion of the maxilla. During this expansion, the maxilla is separated into two parts at the midpalatal suture. This type of expansion, depending on age or growth, the amount of expansion, teeth and the periodontal situation, is carried out in four ways.

##### Conventional Rapid Maxillary Expansion (RME or RPE)

**Figure 18:**
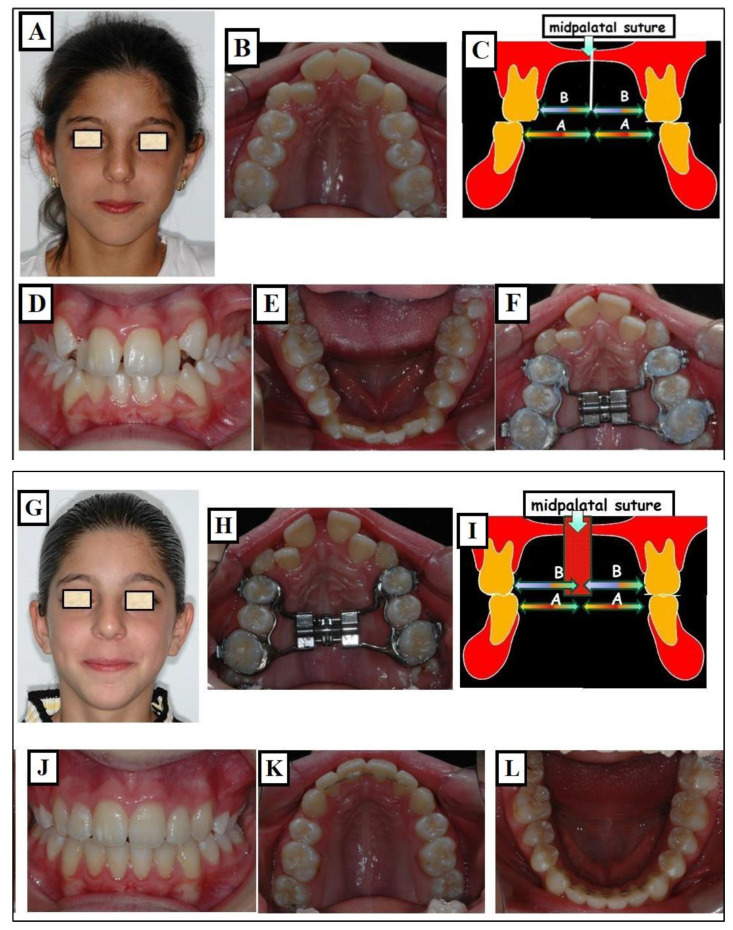
Orthodontic expansion through a conventional RPE (Rapid Palatal Expander). Before treatment, a narrow face and mouth (**A**), narrow upper jaw (**B**), the width of the maxilla as seen from the midpalatal suture (white arrow) is symmetrical and narrow (arrows B) compared to the width of the mandible (arrows A), (**C**), and crossbite (**D**). The lower jaw has adapted to the upper jaw (**E**). An RPE was used for expansion (**F**). (**G**–**L**) describe the status after treatment. After the expansion of the maxilla, there was a harmonization of both jaw widths (arrows A, B) (**I**).

A prevalent therapeutic approach for younger individuals to address maxillary transverse deficiency is Rapid Maxillary Expansion (RME). The objective of this intervention is to broaden the midpalatal suture by exerting lateral forces against the teeth and marginal alveolar bone. RME proves effective in children and adolescents before sutural closure. However, in non-growing adolescents and young adults, the success rate of maxillary expansion decreases with the closure of sutures. (Figure 18A–L).

##### Micro-Implant-Assisted Rapid Palatal Expander (MARPE)

**Figure 19:**
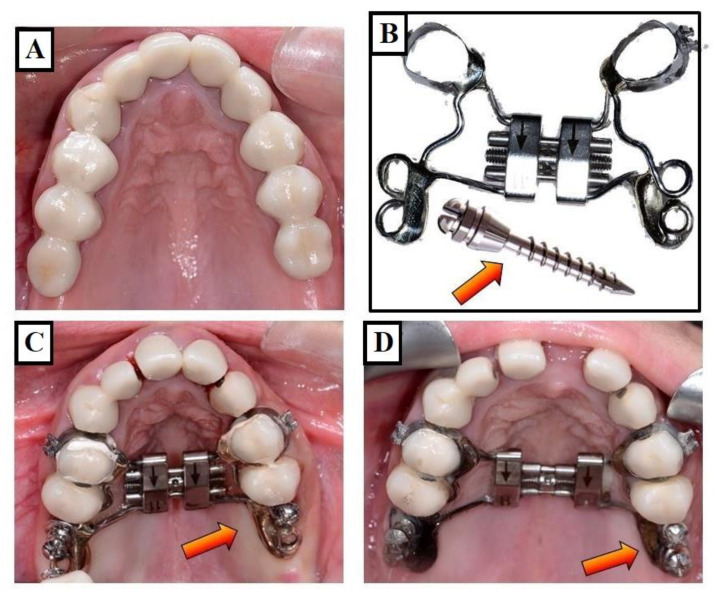
Expansion of the narrow upper jaw (**A**) with the MARPE (Miniscrew-Assisted Rapid Palatal Expander). (**A**–**D**) show the case before expansion, the expansion device in combination with mini-implants (red arrows), before activation of the device, and finally (**D**) after the expansion of the upper jaw, respectively.

In recent years, another palatal expansion design has been developed with a jackscrew attached to the palatal vault by a temporary anchorage device (Figure 19A–D). This design is the micro-implant-assisted rapid palatal expander (MARPE), used to combat undesired dental effects by achieving pure skeletal changes. MARPE is a simple modification of a conventional RPE appliance. The main difference is the incorporation of micro-implants into the palatal jackscrew to ensure expansion of the underlying basal bone, minimizing dentoalveolar tipping and expansion.

##### Surgically Assisted Rapid Palatal Expansion (SARPE)

**Figure 20:**
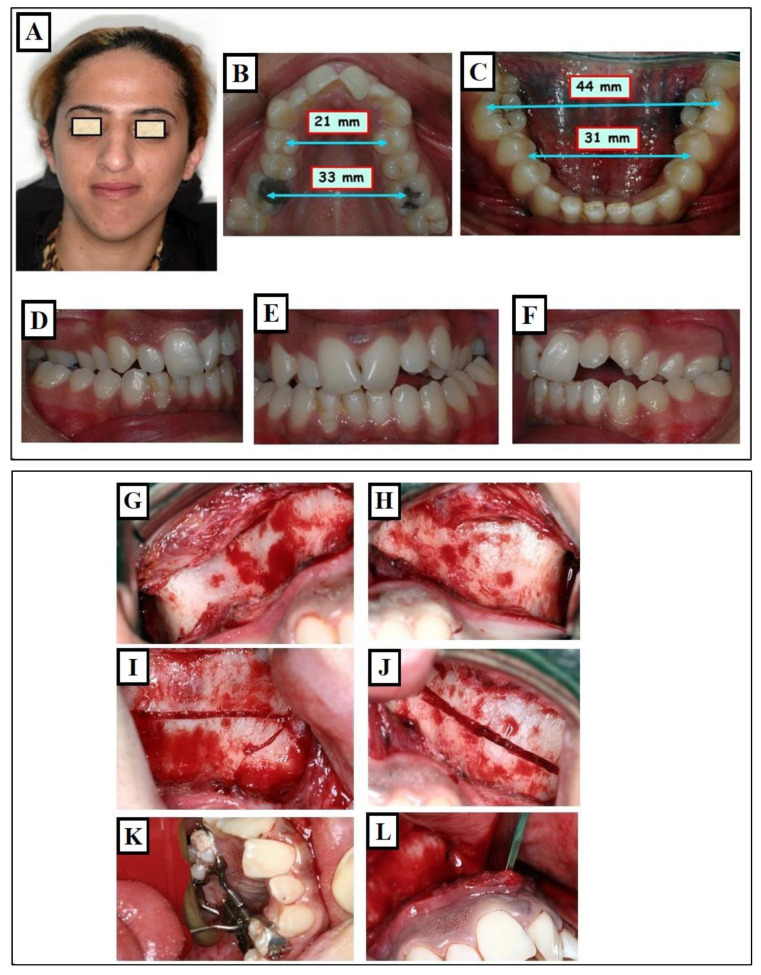

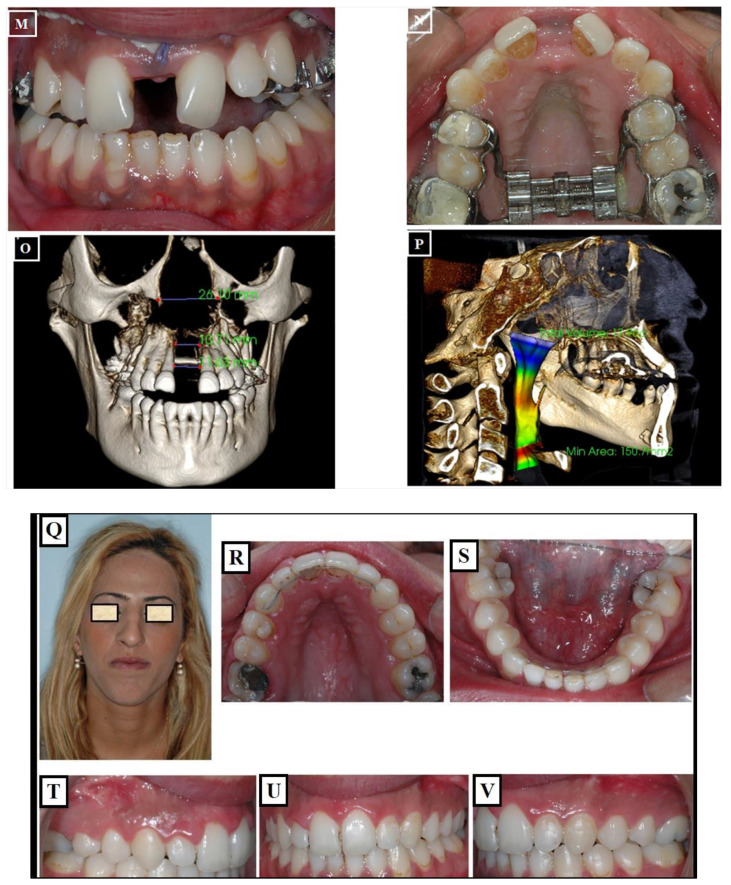
Expansion of the maxilla through Surgically Assisted Rapid Palatal Expansion (SARPE). Situation before treatment: narrow face and narrow nose (**A**), extremely narrow upper jaw (blue arrows 21mm, 33 mm) (**B**) compared to the lower jaw (blue arrows 31mm, 43 mm) (**C**), skeletal and dentoalveolar crossbite on the right and left (**D**–**F**). Surgical assistance for maxillary expansion. Surgical separation of the bones on the maxilla was partially performed at the level of Lefort I (**G**–**L**). Maxillary expansion with the screw (**M**,**N**). Cone-beam computed tomography (CBCT) after expansion shows the separation of Maxillary parts, as part of an examination, the amount of expansion at the bone was measured compared to the screw opening (**O**) and the airway the colored area represents the measured airways before and after expansion (**P**). Improvement in breathing and breathing disorders is expected in such patients. Condition after treatment, changes in facial width (**Q**), changes in jaw widths (**R**,**S**) with occlusion correction (**T**–**V**).

The maturity level of the individual plays a significant role when assessing the impact of Rapid Maxillary Expansion (RME) on craniofacial structures. RME treatment tends to be more effective in children than in adults. Although achieving maxillary expansion in older patients is plausible, the outcomes are not as predictable or enduring. In such instances, surgically assisted RME (SARME or SARPE) is an alternative for adolescents, and for adults, SARME remains the sole option for widening the maxilla. However, complications associated with the surgical procedure and financial constraints limit the widespread applicability of this treatment among adults. The surgical approach might be advisable in patients with extreme maxillary hypoplasia requiring extensive expansion (especially if the posterior teeth incline buccally). It also might be the preferred choice for patients who have significant gingival recession with probable dehiscence and fenestrations, and it might be beneficial for patients with sleep apnea (Figure 20A–V).

##### Absolute Surgically Palatal Expansion (ASPE)

**Figure 21:**
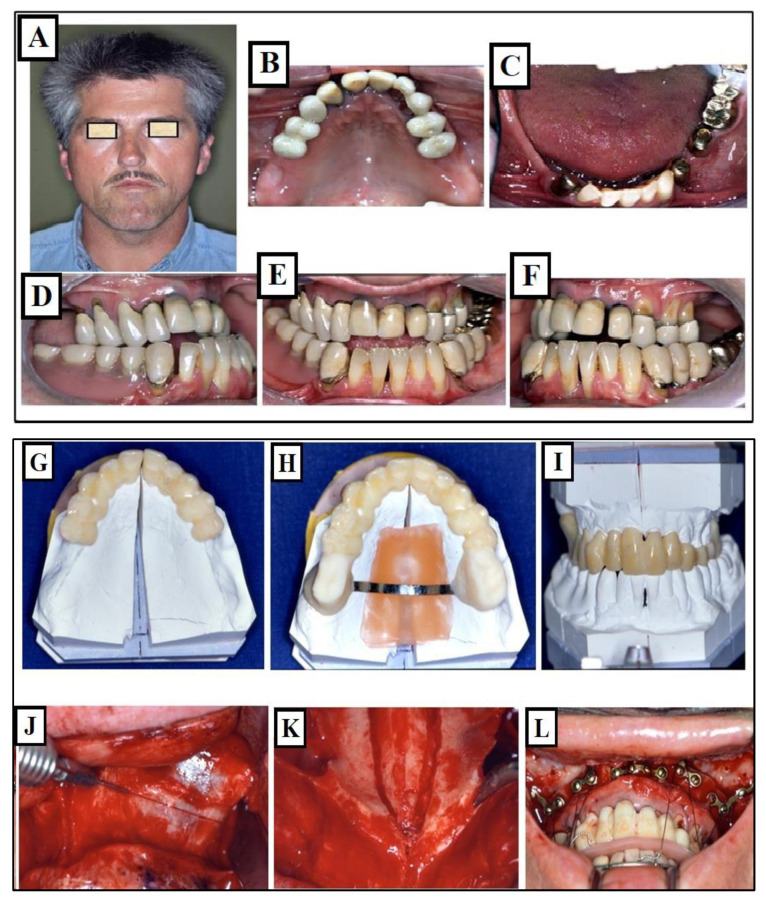

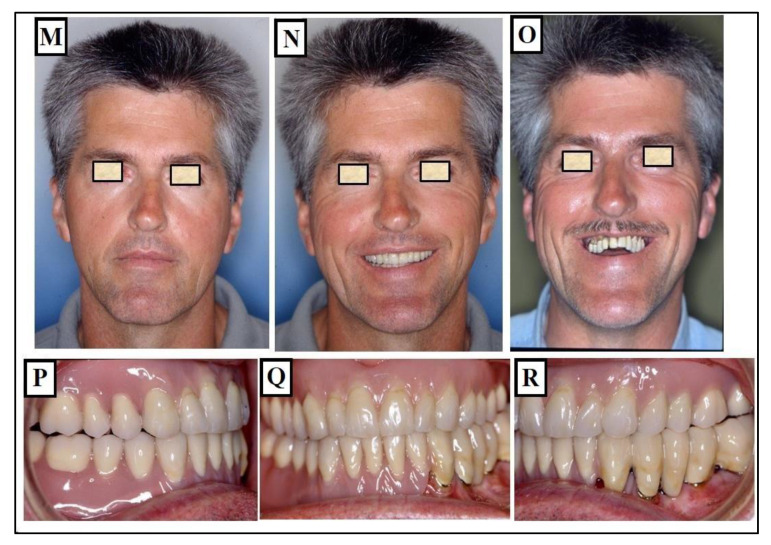
Expansion of the maxilla through Absolute Surgically Palatal Expansion (ASPE). Patient’s situation before treatment, narrow and concave face (**A**), narrow jaws (**B**,**C**) with crossbite on the right and left, and the transverse dimensions (**D**–**F**). Surgical planning of the maxilla on plaster models (**G**–**I**). Absolute surgical expansion and repositioning of the maxilla, paramedian saw cuts were performed for the maxilla (**J**–**L**). The patient after treatment compared to the initial situation (**M**–**O**), correct occlusion in the sagittal, vertical, and transverse dimensions (**P**–**R**).

This involves a surgical separation of the maxilla in the paramedian plane, not within the area of the median palatal suture, for the planned transverse expansion of the maxilla. A preoperative simulation on the surgical models is necessary for this procedure (Figure 21A–R).

This kind of expansion can be achieved by a variety of methods, such as archwire widening, gradual widening, cyclical narrowing and widening, fast widening, and, most recently, growth supported by short-term anchorage devices [42]. For STDs related to mouth breathing, rapid expansion has been utilized. Before beginning orthodontic treatment, it is crucial to address any ongoing causes of STDs that may be detected, such as thumb sucking or mouth breathing.

## 4. Discussion

The dental arch can be effectively widened using a variety of methods. However, it should be emphasized that the amount that any technique can enlarge without also widening the basal bone will be limited. One of the most basic dental expansion tools is an orthodontic bracket with a wide archwire. Quadhelix (Figure 17E) cross-arch elastics, and transpalatal arches (TPA) represent additional instruments [42]. A child should have an orthodontic checkup by the time they are seven years old, in part because of the STD correction recommendation. For example, in individuals classified as class II with an overjet exceeding 7 mm or in class III patients with an overjet less than −1 mm, there are certain skeletal malocclusions that can also be identified early. Any one of these patients could be an STD carrier. It is advantageous to identify and address this shortcoming as soon as possible [42]. Two principal advantages are the uneven development in the case of bilateral crossbite with functional shifts and the decrease or removal of the need for surgical intervention. Some possible secondary advantages include an increased arch circumference to allow for subsequent dental positioning, an improvement in anteroposterior malocclusion, and maybe improved respiratory passage [45]. Orthopedic expansion is easiest to achieve prior to the cranial base and midfacial sutures closing. The lateral dimension, namely at the inter-spheroidal and inter-ethmoidal sutures, has been demonstrated to be the first plane at which growth stops. McNamara and colleagues [46] find that both close before the age of nine. At this stage, up until the latter stages of adolescence, there is limited to negligible skeletal growth. However, during this period, there is a rising emphasis on dental expansion and a declining emphasis on skeletal expansion [47]. The circum-maxillary sutures follow a similar pattern of increased complexity and diminishing patency as they age [48]. A number of methods have been proposed for assessing skeletal maturity: handwrist radiography, cervical vertebral maturation, and, most recently, maxillary sutural maturity assessment using CBCT [48]. Orthopedic expansion appliances are often divided into two categories: fast palatal expanders that are bound to the tooth and tissue, and tooth-plus-tissue borne expanders (Figure 21A–R) or banded to teeth. The more successful of these two has been demonstrated earlier [29,49] and is shown in Figure 18 and Figure 19, while there is still disagreement about which one produces a larger skeleton expansion. In contrast to rapid protocols, which aim for one turn every two to three days, slow activation treatments require one turn every two to three days. Upon completion of the growth period, Proffit et al. [50,51] assert that comparable skeletal or dental expansion occurs. Orthopedic skeletal growth can be indicated by the following: the existence of crossbite; patients in class II and III who are deemed to be not yet reaching skeletal maturity (i.e., still in the growth phase); and patients whose maxillae are narrow [42]. Between 1860 and 1861, Emerson Angell provided the initial accounts of rapid maxillary expansion (RME), which Haas later expanded upon and promoted. RME’s main goal is to enlarge the maxillary arch; however, it also affects ten additional cranial bones in addition to the maxilla [52]. Proponents of RME assert that it produces the least amount of dental movement (tipping) and the greatest amount of skeletal movement [53]. When the posterior teeth are subjected to strong, quick force, the force is transferred to the sutures rather than the teeth over time. The sutures are unsealed, and the teeth move very little in relation to their skeletal support once the appliance is exerting a force greater than what is permitted for orthodontic tooth movement. The mid-palatal suture and ultimately the other maxillary sutures are unsealed, the anchor teeth are tipped, compression of the ligaments connecting the teeth to the jawbone occurs, and bending of the alveolar process occurs [54]. RME appliances can be bonded or banded. For the former, the apparatus is affixed between the upper first molar and the initial premolars utilizing bands. Since the absence of palatal covering, using bonded devices is a better clean and sanitary choice. RMEs with bands could fall into one of a pair of classifications: either dental-borne (e.g., Hyrax widener) or dental- and tissue-borne (e.g., Hass expander) [54]. By increasing the creation of the bone connecting the intermaxillary sutures, slowing maxillary expansion, and potentially removing or reducing RME limits, reduced opposition from tissues is created upon the circummaxillary anatomical elements. Gradual growth has also been found to produce improved post-expansion stability if an appropriate retention period is allowed [55,56,57,58]. This method permits the application of a continuous physiological force until the required expansion is attained. A Quadhelix is a common device for gradual maxillary growth [54]. Dental movement is the only method available for expanding the maxillary dental arch in skeletally mature (non-growing) people. For adult individuals with skeletal transverse deficit, tooth migration beyond the alveolar bone basis of the maxilla may be necessary. This may cause recurrence, perforation of the buccal cortex, upward displacement, root erosion, curvature of the alveolar bone, squeezing of the periodontal membrane, anchor teeth tilting, palatal tissue necrosis, discomfort, and incapacity to commence division of the midpalatal suture and introduce instability in the expansion process [59,60].

Adult patients can alternatively have their skeletal transverse dimensions changed through segmental surgery or surgically assisted orthodontic palatal expansion (SARPE). Traditionally, the main area of resistance to orthodontic extension was thought to be the midpalatal suture. Midpalatal splitting was thus included in the first reports on surgical intervention to support palatal growth [42]. As per Line [61], tf there is not a concurrent vertical and/or horizontal disparity, transverse skeletal deficit is uncommon. If correcting maxillary constriction is the only goal, distraction should be considered. However, widening of the maxillary arch may be a component of a treatment strategy that includes numerous additional orthognathic surgical operations for correction [42]. Segmental osteotomies or SARPE are two surgical techniques that can be used to treat STDs. When treating all maxilla–mandibular abnormalities with a single surgical surgery, the former is favored. This is so that an STD correction can be carried out concurrently with the vertical and sagittal repositioning of the maxilla made possible by segmental osteotomies. On the other hand, with SARPE, maxilla–mandibular realignment in other planes requires a separate surgical procedure after STD correction. Bailey et al. [62] have advocated SARPE for patients with unilateral or asymmetric maxillary constriction, as well as for isolated transverse deficits in patients without any indication of orthopedic maxillary expansion (OME). The enduring consistency, complications, and mental effects in a two-step versus a single-step surgery need to be considered, even though the usage of SARPE may seem limited by this explanation.

The enduring stability and recurrence probability of SARPE have not been thoroughly examined in the literature. Most of the time, surgical expansion is claimed to be more stable over OME [63,64,65,66]. Some writers claim that retention is not required for SARPE, allowing the orthodontist to begin treatment without the need for a holding phase [66]. A post-expansion retention time of two to twelve months is suggested by others in the literature, varying in their recommendations [64,67,68,69,70,71]. Relapse rates from SARPE have been reported to range between 5 and 25% [66,72,73,74]. OME relapse rates can be substantially higher; according to some publications, they can reach up to 63% [75,76,77].

The majority of OME patients are skeletally developed, and the surgery is neither predicted nor permanent for them, which contributes to the elevated incidence of recurrence of OME. In an age-appropriate sample, Berger et al. [73] contrasted SARPE with OME. For OME, respondents ranged in age from six to twelve years, while for SARPE, subjects ranged in age from thirteen to thirty-five years. The stability of the two approaches used in this investigation was not found to differ. For either group, relapse was not measured [36]. The majority of SARPE literature advises clinicians to be mindful of potential relapse; although, it is infrequently reported. There is minimal indication of the necessity for overexpansion in SARPE, although some sources mention it, especially in the context of bone-borne appliances [63,64,78]. In these instances, the occurrence of relapse remained remarkably infrequent [72,79].

In their evaluation of postoperative stability in individuals undergoing orthognathic surgery, Proffit et al. [80] discovered that segmental surgery provides the least amount of stability when it comes to maxillary expansion. But since the evaluation was conducted in 1996, there have clearly been significant advancements in surgical procedures and postoperative control, which have enhanced transverse stability. After tight fixation developed, the same authors did, in fact, publish an improved version of their earlier work in 2007 [51]. Woods et al.’s research [81] revealed that while relapse rates for SARPE were low, segmental surgical procedures exhibited instability, particularly in cases where a significant degree of widening (>8 mm) was required. This review has limitations, including those associated with non-systematic reviews. There is a chance that some pertinent studies were overlooked. However, it seems that there is still a dearth of research on STDs, and additional studies on this subject are required.

In order to gain a deeper comprehension of the mechanisms underlying STDs, the ideal animal model would spontaneously develop STDs in a way akin to that of people. Many novel medications have been developed and assessed using conventional animal models [82,83]. For in vivo research looking at the role of certain genes and genetic changes in acquiring STDs, genetically modified mice are perfect models [84]. They offer early stage models that make it possible to find predictive and correlated biomarkers for evaluating different treatment approaches [85].

The Collaborative Cross (CC) mice model was developed as a consequence of the need to create a high genetic diversity mouse reference population [86,87]. The resource comprises a diverse range of recombinant inbred (RI) lines that were produced from a genetically heterogeneous collection of eight progenitor strains. It is specifically created for the examination of complex traits, suggesting greater power than previously published techniques [88,89]. Due to the evolutionary divergence, the conclusive CC mouse group comprises a notably higher genetic diversity than other mouse models [90,91].

Because of the high number of genetic variations (over 36 million SNPs) that segregate in the population and the relatively high degree of recombination events compared to other mouse populations (4.4 million SSNPs segregate between the founders), the CC mice population is favorable for gene mapping [92,93,94]. Recent research indicates that the quantitative trait locus (QTL) in CC mice typically maps contrast alleles between lines descended from the wild and lines raised in the lab [95,96]. The mapping resolution in the CC population will be less than 1 Mb, according to the activation of QTL analysis. Because of the unique characteristics of the CC panel, it is possible to examine the intricate genetic causes of human disease as well as the interplay between hereditary and environmental factors.

## 5. Conclusions

Assessing STDs is more difficult than evaluating vertical or sagittal differences. In order to effectively treat sexually transmitted diseases, appropriate diagnosis and treatment planning are essential. This ongoing necessity drives the ongoing advancement and modification of diagnostic instruments. Depending on the patient’s growth stage, management approaches can be divided grouped in two primary groups: orthodontic therapy only or a combination of surgical and orthodontic therapy. However, additional research into the stability of each strategy is still required.

## Data Availability

Data are contained within the article.
